# Coping strategies of Nigerian medical students during the COVID-19 pandemic

**DOI:** 10.4314/gmj.v56i1.3

**Published:** 2022-03

**Authors:** OyinOluwa G Adaramola, Oluwaseun M Idowu, Oluwanisola I Toriola, Daniella M Olu-Festus, Toluwanimi E Oyebanji, Christabel I Uche-Orji, Oluseun P Ogunnubi, Oluwakemi O Odukoya

**Affiliations:** 1 College of Medicine, University of Lagos, Nigeria; 2 College of Medicine, University of Ibadan, Nigeria; 3 Department of Psychiatry, College of Medicine, University of Lagos, Nigeria; 4 Department of Community Health and Primary Care, College of Medicine, University of Lagos, Nigeria

**Keywords:** Coping strategies, COVID-19, Medical students, Nigeria

## Abstract

**Objective:**

This study assessed the coping strategies of Nigerian medical students during the COVID-19 pandemic.

**Design:**

We conducted an online descriptive cross-sectional study among medical and dental students attending three of the largest Colleges of Medicine in the Southwestern zone of Nigeria.

**Settings:**

Our study involved students across the pre-clinical and clinical levels of the three Colleges of Medicine.

**Participants:**

We selected the respondents through a purposive sampling technique and disseminated questionnaires applied using an online survey platform (Google forms https://forms.gle/19yfEzehJKwsme759). A total of 1010 participants out of 2404 eligible students completed the questionnaires accurately, giving a response rate of 42%.

**Methods:**

The Brief-COPE questionnaire assessed the participants' coping strategies (approach and avoidant) during the COVID-19 pandemic. We conducted a bivariate analysis using the chi-square test and multiple regression analysis (p< 0.05) to determine the predictors of avoidant coping strategies.

**Results:**

Respondents mean age was 21.8±2.9 years, results were presented as Odds Ratios(OR) at 95% confidence intervals(CI). About 95% of respondents employed an approach coping strategy, while the minority(5%) adopted an avoidant coping strategy. Females were three times more likely to employ an avoidant coping strategy (OR=3.32 (95% CI 1.67–6.21) compared to male students.

**Conclusion:**

This study reveals that the majority of the respondents employed an approach coping strategy towards the COVID-19 pandemic. Females were more likely to employ an avoidant coping strategy. We recommend gender-specific programs to help medical students cope with the COVID-19 pandemic.

**Funding:**

No External Funding

## Introduction

The Coronavirus disease 2019 (COVID-19) pandemic has spread worldwide and created significant obstacles for physicians and medical educators regarding healthcare and medical education delivery.^1^ For medical students, the halt in academic activities could influence their mental health. Fear, worry, and stress are normal responses to perceived threats and uncertainty; thus, in the COVID-19 pandemic, the fear of contracting the virus is an expected response. Medical students may be at risk of psychological stress due to the significant changes in their daily lives brought about by the COVID-19 pandemic, such as lockdowns, social distancing and temporary closure of schools affecting Nigerian students. These events may affect the students' mental health and cause anxiety 2, depression, attrition, and severe health problems.^3^

Coping strategies involve adjusting to or tolerating native events or realities while maintaining a positive self-image and emotional equilibrium.^4^

It is classified into two broad categories: approach and avoidant coping strategies. Approach coping is any behavioural, cognitive, or emotional activity directed toward a threat (e.g., problem-solving or seeking information). Avoidance is any behavioural, cognitive, or emotional action directed away from a threat, for example, denial and withdrawal.^5^

Generally, using more approach and less avoidant coping strategies have been linked to positive outcomes.^5^ Students, may employ coping strategies such as effective time management, social support, positive reappraisal, and engagement in positive pursuits in a bid to reduce stress. However, some of them still struggle with emotional-based strategies like self-blame and self-criticism.^3^

A pre-pandemic study done by Steiner-Hofbauer et al., among undergraduate pharmacy students from a Malaysian public university, reported that some stressors affecting the students were study workload, quizzes, busy work schedules and peer pressure.^6^ The authors of this study further reported the common coping strategies of the students as; sleeping, self-motivation, smoking, positive thinking and the use of motivational quotes.^6^ Another pre-pandemic study among students at the medical university of Vienna showed stressors such as performance pressure overload, social expectations, uncertainty about study, financial uncertainties affecting the medical students.^7^ Some coping strategies adopted by the students in their study include faith/religion, social support, positive thinking and leisure time activities.^7^ A similar study that examined the predictors of academic-related stress and coping among College students at southeastern universities showed that students used emotion-focused coping, problem-focused coping and support from significant others to manage academic stressors.^8^

To illustrate the association of a potential stressor as the COVID-19 pandemic with medical students' perceived stress and coping, Abdulghani et al. reported the burden of online learning and education, anxiety before online learning sessions and difficulty concentrating during online learning sessions as perceived stressors.^9^ Coping strategies demonstrated by these students were; engagement in religious activities, seeking online help and advice from friends and experts, acceptance and learning to live with the COVID-19 pandemic and regular exercises.^9^

In summary, current literature regarding coping mechanisms (whether or not related to the COVID-19 pandemic),^10^–^13^ among students in general in Africa include negative mechanisms such as; problem avoidance, self-criticism, behavioural disengagement, withdrawal, Avoidance, distracting coping, substance use, smoking, crying and overeating. The positive coping mechanisms include; self-encouragement, counselling, praying, emotional support, developing a healthy mind, positive thinking, social support, leisure time activities and preventive action towards hazardous living conditions.

While pre-pandemic studies 6–8 have examined the coping strategies employed by college students, there remains a gap in the literature regarding the coping strategies adopted by medical students during a global pandemic like COVID-19, hence the focus of this study. This study aimed to assess the coping strategies of Nigerian medical students during the COVID-19 pandemic and to assess the relationship between socio-demographic characteristics and coping strategies. Our study provides information on how medical students cope during a major stressor such as the pandemic. This study also hopes to provide empirical evidence to inform the design of coping strategy interventions for medical students and serve as a guide to formulate relevant policies for medical education.

## Methods

A descriptive cross-sectional study was conducted among medical and dental students attending three of the largest Colleges of Medicine in the Southwestern zone of Nigeria; College of Medicine, University of Ibadan (CO-MUI), College of Medicine, University of Lagos (CMUL), Lagos State College of Medicine (LASU-COM). A purposive sampling method was used to select the schools and respondents in the target population. Only students who have completed one full academic session in the selected universities were included in the study. Out of a total population of 2404 eligible students, 1010 filled the questionnaires, giving a response rate of 42%.

### Data collection

Data was collected from the 22^nd^ of June to the 16^th^ of July 2020. An online questionnaire was applied using an online survey platform (Google forms https://forms.gle/19yfEzehJKwsme759) to participants who met the eligibility criteria (all medical and dental students who gained admission into the COMUI, CMUL, and LASUCOM). The online survey link was first disseminated via the WhatsApp social platforms of the university association presidents. They were requested to pass it on to other medical and dental students in the university (through their existing class group chat groups). The structured questionnaires were without names or personal identifiers to ensure the confidentiality and reliability of data. A total of 1010 respondents that completed the questionnaires were included in the final analysis

### Study instrument

The study instrument comprised a structured questionnaire that inquired demographic information and the Brief-COPE (Coping Orientation to Problems Experienced) Questionnaire^14^ (previously developed and used for evaluation of coping strategy in the Chilean adult population) was used to assess the coping strategies of the participants during the COVID-19 pandemic.

The questionnaire contains twenty-eight items that represent the two overarching coping strategies:

- Avoidant coping is characterized by the subscales of denial, substance use, venting, behavioural disengagement, self-distraction, and self-blame. Compared to Approach Coping, Avoidant Coping is less effective at managing stressful events or anxiety.^14^
- Approach Coping is characterized by the subscales of active coping, positive reframing, planning, acceptance, seeking emotional support, and seeking informational support. Approach Coping is associated with more helpful responses to stressful events adversity, including practical adaptive adjustment, better physical health outcomes, and more stable emotional responses.^14^

### Study measures

A four-point Likert-scale (1 = Not at all, 2 = A little bit, 3 = A medium amount, 4 = A lot) were presented for each of the following subscales in the two coping strategies;

- Avoidant Coping subscales include Self-distraction (items 1 and 19), Denial (items 3 and 8), Substance use (items 4 and 11), Behavioral disengagement (items 6 and 16), venting (items 9 and 21), Self-blame (items 13 and 26).^14^
- Approach Coping subscales include Active coping (items 2 and 7), Emotional support (items 5 and 15), Use of informational support (items 10 and 23), Positive reframing (items 12 and 17), Planning (items 14 and 25), acceptance (items 20 and 24).^14^

Humour (items 18 and 28) and Religion (items 22 and 27) are neither Approach nor Avoidance coping.^7^

To identify the predominant coping strategy of participants, the BRIEF COPE sums the scores for all the questions, excluding questions on humour and religion. Scores for Approach and Avoidant subscales are then compared, and participants are placed in the coping strategy category with comparatively higher scores.^14^ Accordingly, the students' scores in both subscales were summed and compared, and participants were assigned a final coping strategy based on their predominantly higher scores.

### Data analysis

We analyzed data using IBM Statistical Package for the Social Sciences (SPSS) Version 21. Continuous variables were presented as mean ± standard deviations, while categorical variables were presented as frequencies and proportions. The Chi square test was used to determine the statistical significance between observed differences for categorical variables. A multivariate regression analysis was done to identify the factors associated with the avoidant coping strategy. The results presented as odds ratios at 95% confidence intervals, at a level of statistical significance of p<0.05. A cut-off p-value of <0.2 was used to include variables into the logistic regression analysis after bivariate analysis (chi-square test).

### Ethics approval and consent participation

Ethical approval was obtained from the Lagos University Teaching Hospital, Health Research Ethics Committee (LUTHHREC), with HREC assigned number: LUTHHREC/EREV/0620/56. Informed consent (online) was obtained from participants before the commencement of the study, and participation was purely voluntary. Respondents who agreed to participate were instructed to check the informed consent box in the online form, indicating that they had read and understood the research purpose and decided to participate. Confidentiality was assured to all respondents, and all information obtained was kept under anonymity. All data obtained is currently safely kept in a password protected database.

## Results

The demographic and selected characteristics of the study population are shown in Table 1. Of the 2404 eligible students, the final study population that completed the questionnaire was 1010 students, resulting in a response rate of 42%. Among the sample of 1010 students, more than half were females 524 (51%), and the mean age of the respondents was 21.8±2.9 years. The majority of the respondents were Christians (85%), single (97%), and of Yoruba ethnicity (73%). The majority of participants (89.9%) had no relatives or acquaintances infected with COVID-19.

**Table 1:** Socio-demographics of the participants

Variables	Frequency n(%) n= 1010
**Age group (years)**	

15–19	220 (21.8)
20–24	650 (64.1)
25–29	111 (11.1)
>30	28 (2.8)
	*Mean ±SD=21.8±2.9*

**Gender**	

Male	486 (48.1)
Female	524 (51.9)

**Ethnicity**	

Yoruba	741 (73.4)
Igbo	176 (17.4)
Other ethnicities*	93 (9.2)

**Religion**	

Christianity	859 (85.0)
Islam	142 (14.1)
Other religions**	9 (0.8)

**Institution**	

COMUI	299 (29.6)
CMUL	410 (40.6)
LASUCOM	301 (29.8)

**Year of study**	

Pre-clinical	410 (40.6)
Clinical	600 (59.4)

**Marital Status**	

Single	989 (97.9)
Married	21 (2.1)

**Family Monthly** **income**	

<N33,000 (<87 USD)	168 (16.6)
N33,001–50,000 (87–132 USD)	143 (14.2)
N50,001–100,000 (132–263 USD)	196 (19.4)
>N100,000 (>263USD)	503 (49.8)

**Previous history** **of a mental illness**	

Yes	29 (2.9)
No	981 (97.1)

**Has a relative/acquaintance** **diagnosed with** **COVID-19**	

Yes	102 (10.1)
No	908 (89.9)

Table 2 shows participants' responses on how they coped during the COVID-19 pandemic. Some of the approach coping responses from the participants reported that 15.7% of the respondents “turned to work or other activities to take their minds off the COVID-19 pandemic” 17% said they “tried to see the COVID-19 pandemic in a different light”, and 29% tried to “looked for something good in what was happening during the COVID-19 pandemic” *a lot*. Additionally, most respondents (62.1%) said they “accepted the reality of the fact that the COVID-19 pandemic has happened” *a lot.*

**Table 2:** Responses of participants to the Brief COPE questionnaire

Variables	Not at all n (%)	A little bit n(%)	A medium amount n (%)	A lot n (%)
I turned to work or other activities to take my mind off the COVID-19 pandemic	313 (30.9)	373 (36.9)	166 (16.4)	159 (15.7)
I concentrated my efforts on doing something about the COVID-19 pandemic	512 (50.7)	380 (37.6)	89 (8.8)	29 (2.9)
I said to myself the COVID-19 pandemic isn't real	862 (85.3)	126 (12.5)	13 (1.3)	9 (0.9)
I used alcohol or other drugs to make myself feel better	965 (95.5)	27 (2.7)	14 (1.4)	4 (0.4)
I got emotional support from others	349 (34.6)	384 (38.0)	180 (17.8)	97 (9.6)
I gave up trying to deal with the COVID-19 pandemic	542 (53.7)	275 (27.2)	106 (10.5)	87 (8.6)
I took action to make the COVID-19 pandemic effect better	359 (35.5)	415 (41.1)	159 (15.7)	77 (7.6)
I refused to believe that the COVID-19 pandemic has happened	932 (92.3)	54 (5.3)	12 (1.2)	12 (1.2)
I said things to let my unpleasant feelings escape	581 (57.5)	295 (29.2)	91 (9.0)	43 (4.3)
I got help and advice from other people	326 (32.3)	456 (45.1)	142 (14.1)	86 (8.5)
I took alcohol and drugs to help me get through my feelings about the COVID-19 pandemic	970 (96.0)	26 (2.6)	7 (0.7)	7 (0.7)
I tried to see the COVID-19 pandemic in a different light to make it more positive	295 (29.2)	344 (34.1)	199 (19.7)	174 (17.0)
I criticized myself	684 (67.7)	201 (19.9)	72 (7.1)	53 (5.2)
I tried to come up with a strategy about what to do	204 (20.2)	345 (34.2)	245 (24.3)	216 (21.4)
I got comfort and understanding from someone	304 (30.1)	372 (36.8)	191 (18.9)	143 (14.2)
I gave up the attempt to cope with the Pandemic	802 (79.4)	153 (15.1)	33 (3.3)	22 (2.2)
I looked for something good in what was happening	122 (12.1)	337(33.4)	251 (24.9)	300 (29.0)
I made jokes about the COVID-19 pandemic	358 (35.4)	405 (40.1)	140 (13.9)	107 (10.6)
I did other things to think about the COVID-19 pandemic less	179 (17.7)	316 (31.3)	271 (26.8)	244 (24.2)
I accepted the reality of the fact that the COVID-19 pandemic has happened	44 (4.4)	109 (10.8)	230 (22.8)	627 (62.1)
I expressed my negative feelings	299 (29.6)	446 (44.2)	178 (17.6)	87 (8.6)
I tried to find comfort in my religion or spiritual beliefs	152 (15.0)	238 (23.6)	173 (17.1)	447 (44.3)
I tried to get advice or help from other people about what to do	303 (30.0)	431 (42.7)	187 (18.5)	86 (8.8)
I learned to live with the effects of the COVID-19 pandemic	83 (8.2)	331 (32.8)	327 (32.4)	269 (26.6)
I thought hard about what steps to take	182 (18.0)	387 (38.3)	264 (26.1)	177 (17.5)
I blamed myself for things that happened	843 (83.5)	112 (11.1)	37 (3.7)	18 (1.8)
I prayed and meditated	108 (10.7)	315 (31.2)	285 (28.2)	302 (29.9)
I made fun of the COVID-19 pandemic	507 (50.2)	352 (34.9)	83 (8.2)	68 (6.7)

Some of the avoidant coping responses among the respondents include; 0.7% of the respondents who took alcohol and drugs to help them get through their feelings about the COVID-19 pandemic, 8.8% gave up trying to deal with the COVID-19 pandemic. 4.3% said things to let their unpleasant feelings escape during the pandemic.

Coping strategy of participants: Of the total number of participants, the majority 958 (94.9%) had an approach coping strategy while only 52 (5.1%) of them had an avoidant coping strategy.

From Table 3, the bivariate analyses showed a statistically significant association (p<0.05) between some socio-demographic variables and coping strategy: gender (p≤0.001), ethnicity (p ≤0.001), religion (p=0.049) and previous history of mental illness (p<0.001). The majority of the participants (65%) in the age group of 20–24 years had approach coping strategies. However, there was no statistically significant association between age group (p=0.688) and coping strategies.

**Table 3:** Factors associated with Avoidance coping strat-egy among the respondents

	Coping strategy (n= 1010)			
Variables	Avoidance n(%)	Approach n(%)	(x^2^)	p- Value
**Age group** **(years)**				

15–19	14 (6.4)	206 (93.6)	2.263	0.688
20–24	32 (4.9)	618 (95.1)		
25–29	6 (5.4)	106 (94.6)		
>30	0 (0.0)	28 (100.0)		

**Gender**				

Male	13 (2.7)	473 (97.3)	11.737	**<0.001**
Female	39 (7.4)	485 (92.6)		

**Ethnicity**				

Yoruba	40 (5.4)	701 (94.6)	76.405	**<0.001**
Igbo	5 (2.8)	171 (97.2)		
Other ethnicities*	6 (0.7)	56 (90.3)		

**Religion**				

Christianity	41 (4.8)	818 (95.2)	6.032	**0.049**
Islam	9 (6.3)	133 (93.7)		

**Year of study**				

Pre-clinical	23 (5.6)	387 (94.4)	2.936	0.569

**Previous history of a mental illness**				

Yes	3 (10.3)	26 (89.7)	1.651	**<0.001**
No	49 (5.0)	932 (95.0)		

**Has had a known relative/acquaintance diagnosed with COVID-19**				

Yes	6 (5.9)	96 (94.1)	1.125	0.724
No	46 (5.1)	862 (94.9)		

In Table 4, a multivariate analysis was done, and the outcome variable was the avoidant coping strategy. The table shows that for males, female students were 3.22 times more likely to use the Avoidant coping strategy (AOR: 3.22 (95% CI: 1.669–6.210), p<0.001). Participants who practised “other religions” were 12 times more likely to use the Avoidant coping strategy (AOR: 12.40 (95% CI: 2.242–68.487), p=0.004) when compared with those practising Christianity.

**Table 4:** Predictors of avoidant coping strategy among medical students

Variables	Adjusted OR	95% CI	p-value
**Gender**			
Male (Ref)	1		
Female	3.22	1.669–6.210	**<0.001**
**Ethnicity**			
Yoruba (Ref)	1		
Igbo	0.52	0.198–1.340	0.174
Other ethnicities*	1.41	0.597–3.333	0.432
**Religion**			
Christianity (Ref)	1		
Islam	1.54	0.715–3.298	0.272
Other religions**	12.40	2.242–68.487	**0.004**
**Previous history of** **mental illness**			
No (Ref)	1		
Yes	2.21	0.617–7.889	0.224

Abbreviations: OR odds ratio, CI confidence interval, (Ref) Reference variable

## Discussion

In this study, the majority (94.9%) of the respondents adopted the approach coping strategy (a positive response) relative to the avoidant coping strategy (5.1%), a negative coping response. The finding is consistent with a similar study carried out among students in the College of Teachers Education, University of Mindanao, Philippines, where a majority (90%) of the respondents follow strict personal protective measures as “protective coping strategies” to the COVID-19 pandemic,^15^ which is also a positive response of coping with stress. A study by Savitsky Bella et al. reported that about 2% of the respondents used alcohol to cope with stressful situations such as the COVID-19 pandemic. This finding agrees with our study, where 0.7% of the medical students said they “used alcohol or other drugs to make them feel better about the COVID-19 pandemic.” 16 More so, alcohol consumption has been reportedly associated with high anxiety levels and negative coping strategies.^16^

Findings from this study report that about 5% of the participants exhibited an avoidant coping strategy. This result corroborates a study carried out among selected colleges in Pune, India, revealing that 4.9% of the participants had a low coping strategy (negative response).^17^ In this present study, socio-demographic variables like gender and religion were statistically significant, this finding differs from Pune, India, which found age to be significantly associated with coping strategies.^17^

This study reported that less than half of the respondents (44.3%) said they “tried to find comfort in religion or spiritual beliefs”.

This finding agrees with Abdulghani et al., which reported the “engagement in religious activity” as a coping mechanism of the participants against the COVID-19 pandemic.^9^ Our study is also similar to the findings from students in the college of teacher education, the University of Mindanao, Philippines.^15^ Their results revealed that 39.1% pray, worship, and study the bible as a coping strategy during the COVID-19 pandemic.^15^ Reports also support the notion that religious and spiritual beliefs play an essential role in coping with mental health conditions.^15^ Previous studies have found that religion makes coping with various personal and collective stressors such as illness, the loss of a child, loss of a job, and war easier. 16, 18

This current study showed that the male gender has a higher approach (positive) coping strategy than females. This finding contradicts previous studies, 17, 19–20, showing females had a higher positive coping style score. An explanation for this difference is found in studies that discovered that women tend to use coping strategies to mask their emotional struggles during stressful conditions, which is less effective. 21–23 Unlike men, who use problem-focused or instrumental methods to handle stressful experiences.

In this survey, the year of study of the participants were not associated significantly with the coping strategies of the respondents. This is contrary to research carried out among nursing students in selected colleges in Pune, India, where the year of study was statistically significant. The first-year students adopted maximum coping strategies, followed by the third year and least by the fourth-year students.^17^ The reason why the level of study (in our research) was not significant may be due to the diversity in the population between the studies.

Based on our study findings, it may be implied that the pandemic contributed to the “avoidant coping strategies” of the medical students. In the context of the economic status of Nigeria, as with several other countries, the increased rate of job loss, reduction in salaries, the shut-down of business firms for reasons related to the pandemic may have predisposed individuals to negative coping strategies. A high percentage of households reported the loss of income since mid-March 2020, as 79% of households reported that their total income decreased.^24^ This reflects in the current study, as lower family income was significantly associated with an avoidant coping strategy.

The pandemic was also characterized by low access to basic needs, social services, medical care, and mental health services, which could lead to anxiety disorders. A survey revealed that many households could not afford needs such as staple foods, soap and cleaning supplies, and access to treatment.^24^ The Association of Psychiatrists in Nigeria have reported that the COVID-19 pandemic has further pushed the prevalence rate of mental illness from 25 to 40 per cent; this could predispose the affected individuals to “avoidant coping strategies” like the use of alcohol and drugs.^25^ Also, the educational sector (including medical education) suffered from the adverse effects of the pandemic as there was the closure of schools and tertiary institutes with no or little access to online education. This may have led to students' engagement in illicit acts and substance abuse during their free time, as participants in our study used alcohol consumption and substance to cope with the pandemic.

### Practical implications

The university should establish gender-sensitive programs for students to improve coping strategies during this and future pandemics. For example, they established support groups for females while teaching males how to develop problem-solving skills. General knowledge on the factors associated with “avoidant coping strategies” will be useful in aiding the university management to formulate policies that would ensure emotional wellbeing as part of health-promoting school initiatives. Additionally, online-based mental health and counselling services for students be established to improve their ability to cope with stressful events, e.g. the pandemic and study workload.

### Limitations

This study has some limitations, and the findings should be interpreted with some caution. There was a possibility of recall and selection bias since data collection was online. Furthermore, the results cannot be generalized to the entire country because it was limited to participants in only three universities in the Southwestern zone of Nigeria. Additionally, the findings in this study do not provide direct causality between independent and coping strategies. Also, the coping strategy tool (Brief-COPE) was not externally validated in the context of this study (although it has been used and validated in previous Nigerian studies).^26^–^29^ Lastly, we did not explore the specific types of stressors the students faced for the coping mechanisms reported in this paper. Future studies should explore the role of specific stressors as contributory factors to poor coping strategies among students in medical schools. Studies with a more representative sample may also be considered to provide generalizable findings to the Nigerian or West African medical student population.

## Conclusion

Most of the respondents adopted an approach coping strategy, while only a few had an avoidant coping strategy towards the COVID-19 pandemic. Since the female participants were three times more likely to exhibit an avoidant coping strategy, we recommended that gender-sensitive coping programs to help students cope during the pandemic be established
